# Maize Biofortification Approaches for Fighting Malnutrition in Sub‐Saharan Africa

**DOI:** 10.1002/pei3.70188

**Published:** 2026-07-15

**Authors:** Bitew Tilahun Engida, Tesfaye Walle Mekonnen, Maryke Labuschagne

**Affiliations:** ^1^ Ethiopian Institute of Agricultural Research Addis Ababa Ethiopia; ^2^ Department of Plant Sciences University of the Free State Bloemfontein South Africa

**Keywords:** biofortification, malnutrition, minerals, provitamin A maize, quality protein maize

## Abstract

Maize is a staple food in sub‐Saharan Africa (SSA); however, commonly used maize varieties are deficient in essential nutrients, including lysine, tryptophan, vitamin A, and minerals such as iron (Fe) and zinc (Zn). As a result, millions of people in this region suffer from one or more nutrient deficiencies that commonly lead to malnutrition‐related diseases. Especially children and nursing mothers are seriously affected. Over 32% of children under 5 years of age are stunted, and 37% of women aged 15–49 years are affected by anemia in SSA. These indicate that the severity of malnutrition remains high in low‐ and middle‐income countries (LMICs), underscoring the need for further work to address this issue. Industrial fortification, clinical supplementation, dietary diversification, and biofortification are strategies to combat malnutrition. Biofortification is one of the most effective and economically viable strategies for improving nutrition in LMICs. However, easily accessing biofortified maize varieties, lack of awareness about the nutritional benefits of biofortified maize among smallholder farmers and consumers, as well as the inadequate availability of biofortified germplasm are some of them among several challenges in SSA. Hence, this review paper focuses on the impact of essential nutrient deficiencies, strategies to combat malnutrition and the techniques to be employed for maize biofortification. It also discusses the challenges faced and offers recommendations for researchers, seed producers, farmers, and policymakers about biofortified maize varieties.

## 1 Introduction

Maize ( *Zea mays* L.) is one of the leading cereal crops grown globally and serves as food, animal feed, and raw materials for various industrial products (Wegary et al. 2019). It accounts for approximately 40% of cereal production in SSA, where over 80% is consumed as food and contributes approximately 30% of the total calorie intake (Ekpa et al. 2019). In this region, consumption rates range from 52 to 450 g/person/day (Prasanna et al. 2021). The high consumption rate of normal maize and lack of dietary diversity cause insufficient levels of essential amino acids, such as lysine and tryptophan; vitamins; and minerals like Fe and Zn, which are vital for well‐being (Prasanna et al. 2020). Consequently, millions of Africans, particularly children and nursing mothers, are at risk of disease related to malnutrition (Rautiainen et al. 2016). Approximately 45% of deaths in children under 5 years of age are due to malnutrition, with 20% classified as stunted and 7.5% classified as underweight in developing countries (Sheoran et al. 2022). Medical supplementation, food fortification, dietary diversification, and biofortification are employed to mitigate malnutrition. These strategies are complementary and can be used together or individually; however, the choice of which to implement depends on factors like resources, accessibility, affordability, sustainability, and technical feasibility (Goredema‐matongera et al. 2021). Biofortification of staple crops, like maize, is a sustainable, cost‐effective, and long‐term strategy for addressing malnutrition, particularly in developing countries (Bouis and Saltzman 2017; Bouis 2018).

Genetic variation in essential nutrient concentrations within maize germplasms is crucial for biofortification (Bouis and Saltzman 2017). Several reports have confirmed the presence of significant genetic variation in maize (Prasanna et al. 2020). Lysine and tryptophan contents in the quality protein maize (QPM) germplasm range from 2.7% to 4.5% and 0.50% to 1.1%, respectively, which are double the conventional ranges of 1.6%–2.6% for lysine and 0.2%–0.6% for tryptophan (Vivek et al. 2008). For a genotype to qualify as QPM, the tryptophan concentration in the kernel endosperm must exceed 0.6% (Kaur et al. 2022). A few maize genotypes enriched with provitamin A (PVA), Zn, and Fe also exceed the breeding target levels: 15 μg g^−1^, 33 mg kg^−1^, and 52 mg kg^−1^, respectively (Bouis and Welch 2010). For example, Menkir et al. (2017) evaluated several inbred lines from 2013 to 2016 that contained up to 23 μg g^−1^ provitamin A. Bänziger and Long (2000) reported Fe concentrations ranging from 9.6 to 63.2 mg kg^−1^ and Zn concentrations ranging from 12.9 to 57.6 mg kg^−1^ in tropical maize germplasms.

There are now significant opportunities to develop maize cultivars that are nutritionally enriched. These opportunities arise from several factors, such as advancements in our understanding of key biochemical pathways involved in metabolite biosynthesis of traits, improved analytical tools for screening germplasms for quality traits, and the potential to use molecular markers and genome editing techniques to accelerate product development. This review highlights the impact of macro and micronutrient deficiency and the consequences on health. We discuss the effectiveness of different biofortification approaches like hybridization, mutation, marker‐assisted selection (MAS), quantitative trait locus (QTL) mapping, genome‐wide association studies (GWAS), genomic selection, genome editing, and genetic engineering to develop nutritionally enriched maize varieties, which can significantly improve dietary quality in vulnerable populations, particularly in rural areas of developing countries, where cereal‐based diets are the mainstay.

## 2 Impact of Essential Nutrient Deficiency

Diets that predominantly depend on maize and other cereals often show significant deficiencies in essential nutrients, including lysine, tryptophan, PVA, Fe, and Zn (Pixley et al. 2011), leading to malnutrition. This includes both protein‐energy malnutrition (PEM) and micronutrient deficiencies, which are significant public health issues in developing countries, particularly in southern Asia and SSA (Kiran et al. 2022). PEM arises from insufficient energy and protein intake, affecting children by causing underweight, stunting, and wasting. Severe forms include kwashiorkor and marasmus (Ahmed et al. 2020). Inadequate intake of vitamins and minerals, often referred to as micronutrient deficiency, is sometimes called “hidden hunger” and affects over two billion people, with over half of global cases found in SSA (Van Der Straeten et al. 2020; Ohanenye et al. 2021). Vitamin A and Zn deficiencies are among the top 10 risk factors for Disability‐Adjusted Life Years (DALYs) in low‐income countries (Table 1). Vitamin A deficiency (VAD) can impair vision and increase infection risk, significantly affecting children and pregnant women (World Health Organization (WHO) 2009). Children under 5 years and women of childbearing age are at the highest risk, and about 33% of children suffer from VAD (World Health Organization (WHO) 2009). In addition, about 17.3% of the world's population is at risk of inadequate Zn intake, and nearly 30% suffer from Fe‐deficiency anemia, which can affect cognitive development and overall health (Wessells and Brown 2012; Kumar et al. 2022). In general, currently, in SSA, about 32% of children under 5 years of age are stunted and 37% of women aged 15–49 years are affected by anemia (FAOSTAT 2026). To combat malnutrition among impoverished populations, developing high‐yielding and nutrient‐rich staple crops like maize is essential (Kiran et al. 2022).

**TABLE 1:** Ranking of the top ten leading risk factor causes of Disability Adjusted Life Years (DALYs) in low‐income countries^a^ (WHO 2009).

No.	Risk factor	DALYs (millions)
1	Childhood underweight	82
2	Unsafe water, sanitation, and hygiene	53
3	Unsafe sex	52
4	Suboptimal breastfeeding	34
5	Indoor smoke from solid fuels	33
6	Vitamin A deficiency	20
7	High blood pressure	18
8	Alcohol use	18
9	High blood glucose	16
10	Zinc deficiency	14

^a^ Countries grouped by 2004 gross national income per capita: low income (US$ 825 or less).

## 3 Strategies to Combat Malnutrition

**FIGURE 1:**
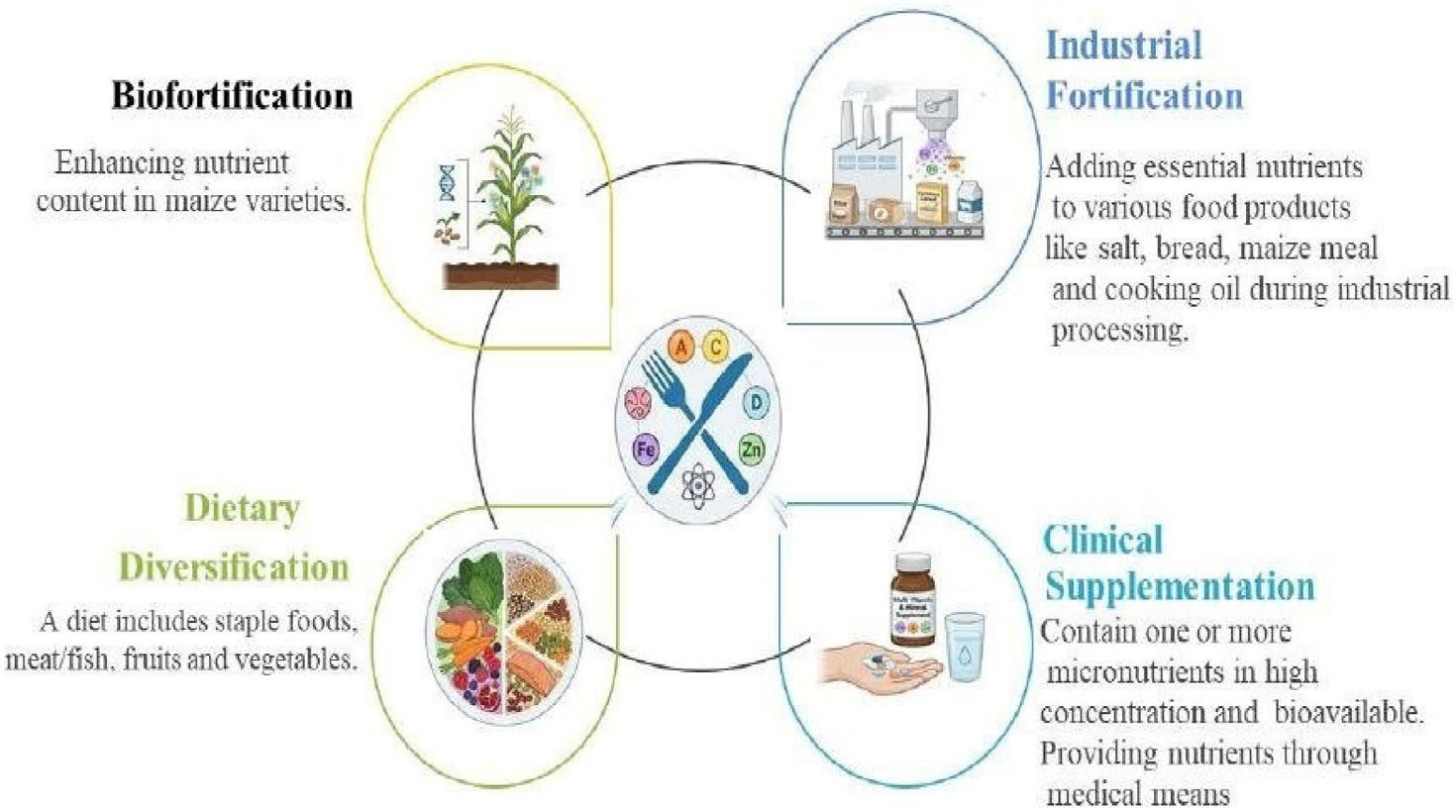
Different strategies to enhance essential nutrient intake in the diet. Adapted from Maqbool and Beshir (2019), Goredema‐matongera et al. (2021) and Kiran et al. (2022). The figure was created by the authors using FigureLabs.ai, but a little modification has been done on it using PowerPoint.

Several strategies have been implemented to increase the consumption of essential nutrients in the diet. These strategies include industrial fortification, clinical or pharmaceutical supplementation, dietary diversification, and biofortification (Figure 1). These strategies are complementary and can be used together or individually; however, the choice of which to implement depends on factors like resources, accessibility, affordability, sustainability, and technical feasibility (Goredema‐matongera et al. 2021). Taking into account these parameters, biofortification of staple crops such as maize is essential for providing nutrients to combat malnutrition‐related diseases in the long term at a cost‐effective rate in SSA.

### 3.1 Industrial Food Fortification

Food fortification is the addition of essential nutrients to various food products during industrial processing (Maqbool and Beshir 2019). In SSA, many countries, including South Africa, Zimbabwe, Nigeria, Uganda, Malawi, and Kenya, have implemented food fortification programs for staple foods (Goredema‐matongera et al. 2021). This strategy involves fortifying basic commodities such as salt, bread, maize meal, and cooking oil with vitamins like A and minerals such as iodine, Zn, and Fe (Eichler et al. 2012). Most of the time, the health sector has often used fortified foods to address nutritional issues among at‐risk groups, including infants, mothers, the elderly, and individuals with chronic diseases or acute nutritional deficits in humanitarian contexts (IFC 2024). However, food fortification has its own limitations, including potential health risks due to toxicity when added above recommended nutrient levels (Hess and Brown 2009) and less availability and may increase the cost of food for low‐income populations in developing countries (Ohanenye et al. 2021). In addition, reports on bread flour and maize meal in South Africa indicate that some food processors skimp on required nutrient levels to cut costs, leading to inadequate fortification (Yusufali et al. 2012).

### 3.2 Dietary Supplementation

Dietary supplementation programmes are mainly initiated for target populations at higher risk of nutritional deficiencies (Maqbool and Beshir 2019). For example, Zn supplements include Zn sulphate (ZnSO4), Zn acetate, Zn gluconate, and Zn oxide (ZnO) are recommended in varying doses by age: 5 mg/day for infants under 36 months, 10 mg/day for older children (Goredema‐matongera et al. 2021). Vitamin supplements, including specific vitamins and multivitamins, are also common. In many developing countries, children receive vitamin A supplements in the form of capsules every 6 months. These capsules contain retinol, which the liver stores and releases gradually to meet vitamin A needs for four to 6 months (Groote et al. 2011).

### 3.3 Dietary Diversification

Dietary diversification is the consumption of a variety of foods that contain essential nutrient‐rich plant and animal products to meet the nutritional demands of populations. A diverse diet helps reduce the risk of both macro and micronutrient deficiencies and serves as a practical long‐term solution. A study conducted on children aged 6–23 months in Tanzania found that consuming a diverse diet significantly decreased the rates of stunting, wasting, and being underweight (Khamis et al. 2019). Animal foods like red meat, fish, and eggs are rich in zinc, bioavailable calcium, iron, vitamin A, and essential fatty acids (Aakre et al. 2020). For instance, 100 g of fish in the diet would contribute substantially to the recommended nutrient intake for vitamin B12, vitamin D, and vitamin A, as well as the minerals iodine (I), Zn, and calcium (Ca) (Aakre et al. 2020). Plant‐based foods such as legumes, fruits, and vegetables also provide substantial minerals and essential amino acids. However, the seasonal availability of some nutrient‐rich fruits and vegetables further complicates access (Ortiz‐Covarrubias et al. 2019). The affordability of animal‐source foods (ASFs) is also challenging, especially in LMICs and among low‐income populations in high‐income countries (The Global Alliance for Improved Nutrition (GAIN) 2020). Establishing diversified nutrient farms could improve food access for poor communities, but this requires government support through agricultural extension services (Goredema‐matongera et al. 2021).

### 3.4 Biofortification

Biofortification is a technique that harnesses the power of plant breeding to enrich staple crops with essential amino acids, vitamins, and minerals (Bouis et al. 2011). Several studies have been conducted to determine the cost‐effectiveness of biofortification. An analysis conducted by the World Food Program (WFP) indicated that in Punjab Province of Pakistan, providing households with biofortified zinc wheat could lead to a 12.6% reduction in the cost of a nutritious diet for rural residents and an 11.5% reduction for urban residents. The cost of a healthy diet for adolescent girls in Punjab is expected to decrease by 26% in rural areas and by 25% in urban areas (Bouis et al. 2024). Resilience is a key benefit highlighted by the growth of biofortification during significant disruptions to food systems, such as the COVID‐19 pandemic and other crises (Bouis et al. 2024).

Biofortification is still an emerging technology used to address micronutrient deficiencies in LMICs (Bouis and Saltzman 2017). However, most biofortification programs have focused on single‐nutrient solutions, which may not address the integrated nutrient deficiency challenges in SSA (Goredema‐matongera et al. 2021). Therefore, most researchers suggested multi‐nutrient biofortification strategies for varieties that already have preferred agronomic and consumption traits, such as high yields (Bouis 2018; Goredema‐matongera et al. 2021). Produced and marketed biofortified crops in surplus amounts may make their way into retail outlets, reaching consumers first in rural areas and then in urban areas (Bouis et al. 2013). For the last two decades, improving the nutritional quality of maize has been a major objective of national and international breeding programs. Therefore, the development, testing across locations, and distribution of QPMs, PVAs, and Zn‐enriched maize in SSA, Asia, and Latin America have advanced significantly (Maqbool and Beshir 2019; Prasanna et al. 2020).

## 4 Essential Nutrients Used in Maize Biofortification

### 4.1 Quality Protein Maize

The maize kernel has approximately 82% endosperm, 12% germ, and 6% pericarp (Prasanna et al. 2001). The endosperm is the primary source of carbohydrates; however, 80% of the protein content is also present in the endosperm, with the remaining protein located in the germ. Maize endosperm proteins are categorized into four classes based on their solubility: albumins, which are soluble in water; globulins, which are soluble in saline solutions; prolamins (zeins), which are soluble in alcohol; and glutelins, which are soluble in alkali solutions (Maqbool et al. 2021). The average protein fraction distribution in the maize endosperm is 34% glutelins, 60% prolamins/zeins, 3% albumins, and 3% globulins (Vasal 2000). All the fractions except zein are well balanced and have high levels of lysine and tryptophan (Vasal 2000). Although zein proteins are low in the essential amino acids, lysine and tryptophan, they are rich in glutamine (21%–26%), leucine (20%), proline (10%), and alanine (10%) (Sofi et al. 2009; Maqbool et al. 2021). A lack of lysine and tryptophan and an excess of leucine are indications of poor protein quality (Sethi et al. 2021).

In the mid‐1960s, following the discovery of the naturally occurring mutant gene *opaque 2 (o2)*, scientists made considerable efforts to increase the nutritional quality of the maize endosperm protein (Vasal 2000). According to Teklewold et al. (2017), the *o2* gene contains double the content of lysine and tryptophan in the endosperm compared with commonly used maize genotypes. This advancement could increase the protein content of maize, which may improve its nutritional value, especially in developing nations (Wegary et al. 2011). Finally, the QPM germplasm was developed through extensive manipulation of the *o2* gene, resulting in homozygous recessive *(o2o2)* genes, which differ from the homozygous dominant *(O2O2)* genes found in conventional maize (Twumasi‐Afriyie et al. 2016). The development of QPM involves manipulating of three genetic systems: the first is to ensure the presence of the recessive mutant allele of the *o2* gene, the second is to confer the modifier/enhancer of *o2* containing high lysine and tryptophan, and the third is to ensure the *o2* modifier gene converts soft endosperm to hard endosperm (Maqbool et al. 2021). As a result, QPM genotypes can produce 70%–100% higher tryptophan concentration compared to the normal maize varieties (Amegbor et al. 2022; Drochioiu et al. 2024). In addition to *o2*, several opaque mutants (*o1*, *o5*, *o9*–*11*, *o13*, *016*, and *o17*) and floury mutants (*fl‐1*, *fl‐2*, and *fl‐3*) were discovered (Maqbool et al. 2021). Among these, the *o16* gene had a great contribution to enhance the lysine and tryptophan content in maize endosperm (Sarika et al. 2018; Wang et al. 2019). For instance, the recessive *o16o16* seed endosperm was found to be vitreous phenotypically and grain hardness was comparable with that of the normal and QPM maize (Sarika et al. 2018).

CIMMYT has been the primary source of global QPM germplasm, developing QPM varieties (Tumasi‐Afriyie et al. 2011). A few QPM populations and pools having different maturity groups, grain colors, and textures adapted to different ecological conditions have been developed (Prasanna et al. 2001). More than 167 QPM varieties have been released and deployed worldwide, but more than half (53%) of the total QPM varietal releases have occurred in Africa (Twumasi‐Afriyie et al. 2016). Some of them are listed in Table 2. The adoption of Quality Protein Maize (QPM) has been limited, though Ghana led its adoption in 1992, followed by countries like Uganda, Ethiopia, Tanzania, Kenya, South Africa, Burkina Faso, Mali, Mozambique, Malawi, Nigeria, and Zimbabwe (De Groote et al. 2016; Twumasi‐Afriyie et al. 2016). By 2015, approximately 1 million hectares in Sub‐Saharan Africa were cultivated with QPM, mainly in Ghana and Uganda together accounting for ~50% of this. This success stems from QPM's superior agronomic performance, nutritional benefits, and strong government support for its promotion and dissemination (Twumasi‐Afriyie et al. 2016).

**TABLE 2:** List of QPM maize varieties released from 2010 to 2015 in some African countries (Twumasi‐Afriyie et al. 2016).

Country	Variety designation	Variety type	Area of adaptation	Year of release
Ethiopia	AMH760Q	Hybrid	Highland	2011
Ethiopia	MHQ138	Hybrid	Low moisture stress and moist mid‐altitude	2012
Ethiopia	Melkasa‐1Q	OPV	Low moisture stress	2013
Ethiopia	BHQPY548	Hybrid	Moist mid‐altitude	2015
Tanzania	NATAH6Q	OPV	Mid altitude	2013
Tanzania	MAMS H0913	Hybrid	Mid altitude	2014
Uganda	VP Max	OPV	Mid altitude	2012
South Africa	QS‐Mini	OPV	Mid altitude	2010
South Africa	Nelson's choice QPM	OPV	Mid altitude	2012
South Africa	CAP9006QS	Hybrid	Wet & dry mid‐altitude	2012
South Africa	CAP9444NG	Hybrid	Wet & dry mid‐altitude	2013
South Africa	CAP9015	Hybrid	Wet & dry mid‐altitude	2014
South Africa	Q623	Hybrid	Wet & dry mid‐altitude	2014
South Africa	SA4115Q	Hybrid	Wet & dry mid altitude	2015
South Africa	JEMAT601Q	Hybrid	Wet & dry mid altitude	2015
Zambia	GV687P	Hybrid	Mid altitude	2015
Zambia	GV682P	Hybrid	Mid altitude	2015
Zimbabwe	MQ623	Hybrid	Wet & dry mid‐altitude	2014
Lesotho	VP05120	OPV	Wet & dry mid‐altitude	2011
Ghana	Omankwa	OPV	Forest, Forest‐savanna transition, Guinea	2010
Ghana	Abontem	OPV	Forest, Forest‐savanna transition, Guinea	2010
Ghana	Enibi	Hybrid	Forest, Forest‐savanna transition, Guinea	2010
Ghana	Aburohemaa	OPV	Forest, Forest‐savanna transition, Guinea	2010
Nigeria	(Sammaz 14)	OPV	Savanna, Sudan savanna	2010
Nigeria	EV 99 QPM	OPV	Forest, Forest‐savanna transition, Guinea	2010

QPM offers numerous health benefits when it is produced and consumed in sufficient quantity. It provides a more balanced protein source for both humans and monogastric animals, as its protein quality and biological value are significantly greater than those of commonly used maize types (Tandzi et al. 2017). Normal maize protein has a biological value of approximately 40% milk protein; however, QPM protein has a biological value of 90% milk protein (Nuss and Tanumihardjo 2011), which is close to the nutritional value of milk protein. Efficacy studies conducted with QMP have shown its nutritional benefits for both livestock and humans. Feeding experiments conducted on infants and young children demonstrated a 12% increase in weight gain and a 9% increase in height among those found in mild to moderate undernutrition (Gunaratna et al. 2010). Compared with those of pigs fed normal maize, the growth rate of QPM‐fed pigs also significantly increased, with QPM‐fed pigs growing twice as fast (Vivek et al. 2008). According to the Chinese Academy of Agricultural Science (CAAS), nutritional studies conducted on poultry and pigs showed that QPM is better than normal maize in improving animal growth and performance (Qi et al. 2004). In general, for people who are at risk of malnutrition, such as pregnant women, lactating mothers, and young children, QPM can be an excellent supplementary food (Vivek et al. 2008). In addition, refugees and those experiencing dietary challenges around the world can also benefit from QPM flour (Tandzi et al. 2017).

### 4.2 Provitamin A Maize

**FIGURE 2:**
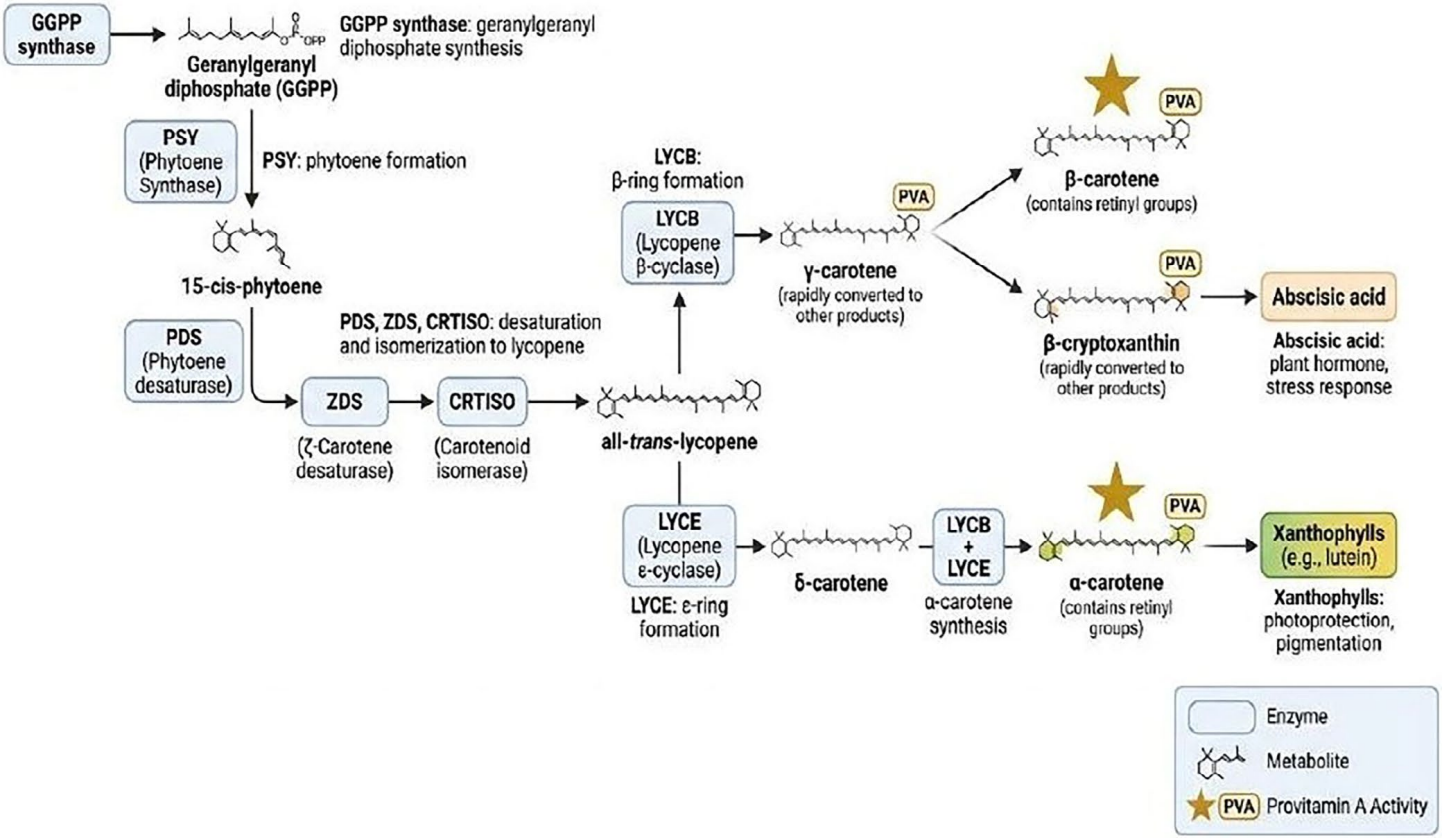
Key steps of the carotenoid biosynthesis pathway in maize. The pathway is adapted from Chen et al. (2010), Farré et al. (2010), and Maqbool et al. (2018). The figure was created by the authors using FigureLabs.ai.

Maize is an essential source of carotenoids. Especially, yellow or orange maize grains have exceptionally high carotenoid concentrations, making them valuable dietary sources (Howitt and Pogson 2006). Understanding the carotenoid biosynthesis pathway in yellow/orange maize kernels is essential for enhancing PVA content. The primary precursors to provitamin A carotenoids are α‐carotene and β‐carotene, which contain retinyl groups and, and γ‐carotene and β‐cryptoxanthin also contribute to PVA levels but quickly convert to other products (Farré et al. 2010). The biosynthetic process begins with the formation of geranylgeranyl diphosphate (GGPP), which is catalyzed by the enzyme GGPP synthase (Figure 2). Two molecules of GGPP then condense to form 15‐cis‐phytoene, catalyzed by phytoene synthase (PSY) (Maqbool et al. 2018). The subsequent desaturation reactions, performed by phytoene desaturase (PDS) and β‐carotene desaturase (ZDS), convert phytoene into all‐trans‐lycopene through carotenoid isomerase (CRTISO) (Chen et al. 2010; Farré et al. 2010). At the lycopene stage, the pathway splits into two branches: one involving lycopene β‐cyclase (LYCB) and the other lycopene ε‐cyclase (LYCE), both of which lead to the formation of various carotenes. LYCB adds two β‐rings to create β‐carotene, which is noted for its superior PVA activity due to its double retinyl groups. The pathway ultimately concludes with the production of xanthophylls (lutein) and abscisic acid (Maqbool et al. 2018).

There has been substantial progress in the global development of maize germplasm to increase provitamin A carotenoid levels. This effort has resulted in the creation of several provitamin A‐enriched maize varieties, which have been successfully introduced and widely consumed in some African countries (Garg et al. 2018). According to HarvestPlus (2023) 78 PVA varieties have been released in various African countries, providing communities with a vital source of micronutrients. Some of them, which were released from 2020 to 2023, have been listed in Table 3. HarvestPlus, which operates under the auspices of the Consultative Group for International Agricultural Research (CGIAR), has dedicated the last two decades to the development and dissemination of micronutrient‐rich staple food crops aimed at improving nutrition and public health globally (HarvestPlus 2023).

**TABLE 3:** List of PVA‐enriched maize varieties released from 2020 to 2023 in some African countries.

Country	Variety name	IITA name	Year released	Yield t/ha	PVA (μg/g)	References
Nigeria	SAMMAZ 59	F2SCA1413–36	2020	4–8	13–16	Gedil et al. (2024)
Nigeria	SAMMAZ 60	F2SCA1413–12	2020	4–7	13–16	Gedil et al. (2024)
Ghana	CRI‐Ewool	LY1409‐21	2021	4–7	9–14	Gedil et al. (2024)
Ghana	CRI‐HarvestPlus	LY1501‐7	2021	5–7	10–13	Gedil et al. (2024)
Ghana	ZAAMS666A	LY1001‐23	2021	5–8	8–10	Gedil et al. (2024)
Ethiopia	BHA5211	LY1001‐23	2022	10	9.0	Tilahun et al. (2024)
Nigeria	HAKIMI 1	LY1606	2022	5–7	11–12	Gedil et al. (2024)
Nigeria	SAMLAK 1608 LY	LY1608	2022	5–7	12–13	Gedil et al. (2024)
Nigeria	SAMMAZ 67	A1820‐4	2022	9.7	NA	HarvestPlus (2023)
Nigeria	WAC42PVEE	EEPVAH‐42	2022	6.2	NA	HarvestPlus (2023)
Nigeria	WAC58PVEE	EEPVAH‐58	2022	7.4	NA	HarvestPlus (2023)
Nigeria	Oba Super 8	EEPVAH‐68	2023	8.3	NA	HarvestPlus (2023).
Uganda	NARO‐maize‐63‐VitA	PVAUG‐2	2023	5.7–7.7	11.32	HarvestPlus (2023)

Abbreviations: IITA, International Institute of Tropical Agriculture; NA, not available; PVA, provitamin A; t/ha, ton per hectare.

Vitamin A is essential for maintaining vision, supporting the immune system, facilitating reproduction, and promoting growth, especially in children and nursing mothers. Studies show that consuming PVA maize can significantly enhance children's vitamin A levels, increasing serum retinol concentrations and improving their ability to see in low light (Palmer et al. 2021; Baudron et al. 2024). Furthermore, breastfeeding mothers who eat PVA maize for 3 months can see over a 50% improvement in the vitamin A concentration of their milk, benefiting both themselves and their infants (van Ginkel and Cherfas 2023; Baudron et al. 2024).

### 4.3 Minerals

Fe and Zn are the most essential minerals that play crucial roles in various biological processes within the human body. Fe is primarily found in red blood cells as a component of hemoglobin, where it functions as an oxygen transporter, delivering oxygen from the lungs to tissues and assisting in oxidative metabolism (Obeagu 2025). Most of the time, Fe is stored in the liver in the form of ferritin and hemosiderin, and the body does not have a system for excreting it (Hurrell and Egli 2010; Abbaspour et al. 2014). It is important for various bodily functions, including enzyme production, hormone regulation, and the development and functioning of the immune and reproductive systems. It plays a crucial role in several metabolic reactions, including energy production, immune defense, and hormone regulation. It is a key component of various heme and nonheme iron enzymes, cytochromes, and ferredoxin electron carriers (Teh et al. 2024). Moreover, Fe is vital for the formation of bile acids and steroid hormones in the liver and helps regulate signals in some neurotransmitter systems, such as serotonin and dopamine in the brain (Kumar et al. 2015).

Zn is important for the function of more than 300 enzymes and plays crucial roles in various metabolic pathways, including those in the central nervous system (Wang et al. 2023). It is also a key component of Zn finger proteins that regulate deoxyribonucleic acid (DNA) transcription (Levenson and Morris 2011). It is important for cell division, growth, immune response, and reproductive functions (Thakur et al. 2015). Overall, Zn is necessary for many biochemical, immunological, and clinical functions (Gibson 2012).

## 5 Maize Biofortification Approaches

**FIGURE 3:**
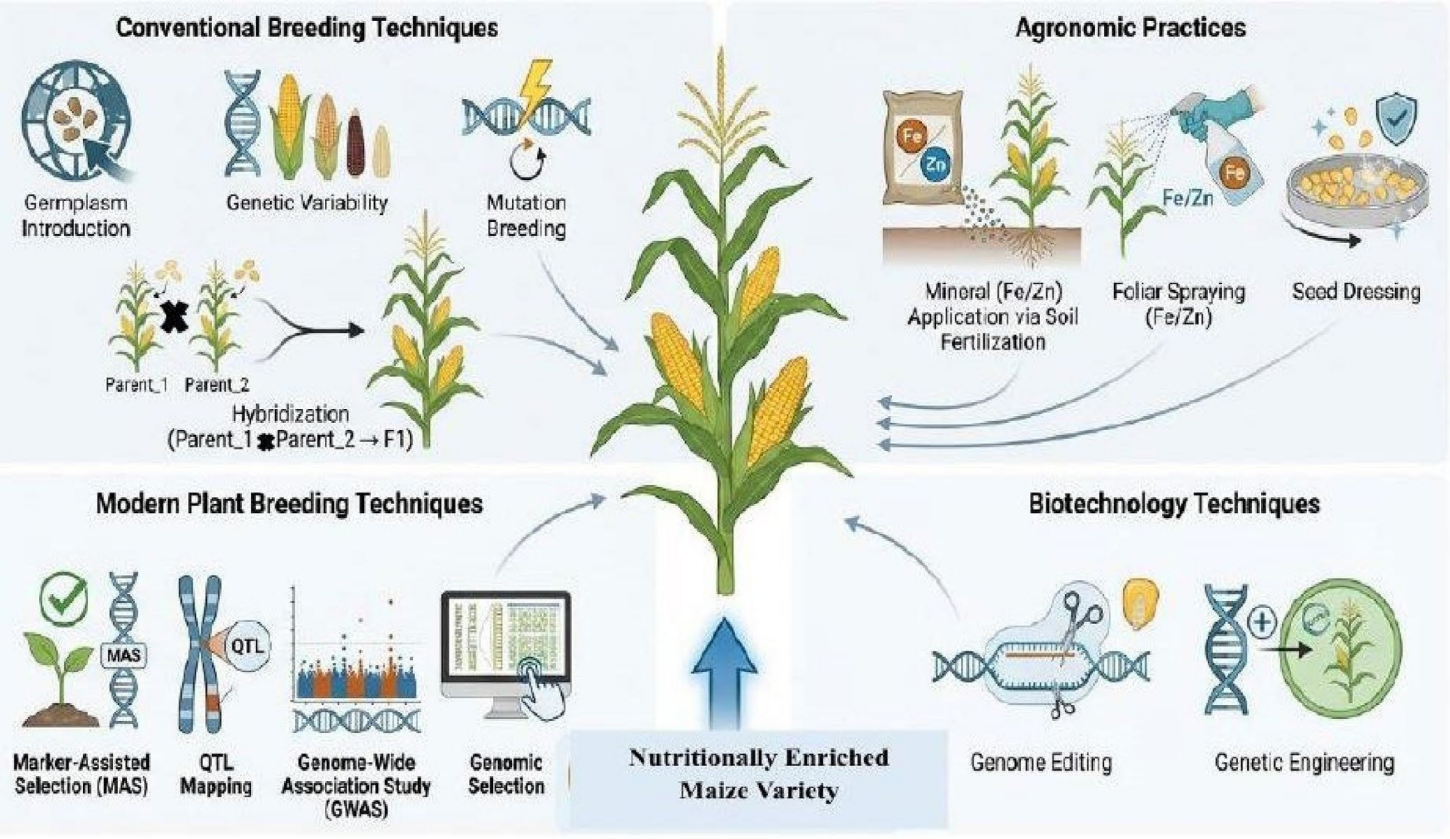
Major maize biofortification approaches for developing nutritionally enriched maize varieties. Germplasm introduction: Introducing nutritionally enriched maize varieties from different regions for adaptation and evaluation. Hybridization: Crossing different maize inbred lines to improve nutritional quality, grain and other agronomic traits. Mutation breeding: Inducing genetic variation using mutagens. Agronomic techniques, such as foliar spray, soil and seed dressing of micronutrients, result in greater nutrient accumulation in edible parts of the plants, delivering healthier foods to the consumer. Marker‐assisted selection: Selecting traits using genetic markers. Quantitative trait loci mapping: Identifying specific regions in chromosomes associated with the target traits. Genomic selection: Predicting genotype performance using genome‐wide markers. Genome editing: Precisely modifying specific DNA sequences to improve nutritional quality traits in maize. Genome engineering: Introducing genes from other organisms into maize to improve the trait of interest. The figure is adapted from Goredema‐matongera et al. (2021) and Maqbool et al. (2021). The figure was created by the authors using FigureLabs.ai.

Nutritionally enriched maize varieties can be developed through conventional, nonconventional, and integrated breeding approaches (Maqbool and Beshir 2019; Goredema‐matongera et al. 2021). Agronomic practices, introductions, hybridization, mutation, marker‐assisted selection (MAS), quantitative trait locus (QTL) mapping, genome‐wide association studies (GWAS), genomic selection, genome editing, and genetic engineering are the major techniques used for the biofortification of staple crops (Figure 3). Some of the commonly used and potential breeding methods are discussed below.

### 5.1 Genetic Variability

Confirming the existence of sufficient genetic variability among germplasms for the target nutrient concentration is the primary requirement for making genetic improvements through breeding (Maqbool and Beshir 2019; Goredema‐matongera et al. 2021). Genetic variability analysis is crucial for identifying cultivars with the best nutritional quality traits. Interestingly, maize exhibits significant genetic variability in several nutritional quality traits (Menkir 2008). Genetic differences in a population are often due to the presence of various alleles of a particular gene. Some beneficial alleles associated with nutritional traits are commonly found in landraces, wild relatives, and improved cultivars grown across the world. Nutritional traits, such as phlobaphenes in red maize, anthocyanins in blue, black, and purple maize, carotenoids in orange and yellow maize, and essential minerals such as Zn and Fe, vary among the landraces preserved at the CIMMYT gene bank (Prasanna et al. 2020). These landraces serve as valuable genetic resources that breeders from both national and international research programs can utilize.

Several studies have demonstrated considerable genetic variation in the concentrations of Fe, Zn, and PVA. The variations in Fe and Zn concentrations suggest the potential to achieve the target levels established by the HarvestPlus program for maize. These targets are 52 μg g^−1^ for Fe, 33 μg g^−1^ for Zn, and 15 μg g^−1^ for PVA (Bouis and Welch 2010). Bänziger and Long (2000) reported Fe concentrations ranging from 9.6 to 63.2 mg kg^−1^ and Zn concentrations ranging from 12.9 to 57.6 mg kg^−1^ in tropical maize germplasms. Economically important differences were also recorded in studies conducted to determine the provitamin A contents in tropical yellow maize genotypes. Menkir et al. (2017) reported that some inbred lines evaluated between 2013 and 2016 contained up to 23 μg g^−1^ provitamin A content, whereas Udo et al. (2023) reported Zn, Fe, and PVA values ranging from 22.51 to 33.33 mg kg^−1^, 20.04 to 29.65 mg kg^−1^, and 3.55 to 15.28 μg g^−1^ contents in maize hybrids, respectively. Studies assessing essential amino acids, particularly lysine and tryptophan, revealed variability among QPM genotypes (Wegary et al. 2011; Ngaboyison et al. 2012). Some genetic variability studies conducted on tryptophan, provitamin A, Fe, and Zn concentrations in maize genotypes are presented in Table 4.

**TABLE 4:** Genetic variability in maize grain for tryptophan, provitamin A, Fe, and Zn concentration.

Traits	Analytical method	Concentration	Genotypes used	References
Tryptophan (g kg^−1^)	Colorimetric	0.50–1.03	108 F1 QPM hybrids	Wegary et al. (2011)
Tryptophan (%)	Colorimetric	0.08–0.12	42 QPM hybrids	Ngaboyison et al. (2012)
	Spectrophotometric	0.03–0.09	40 inbred lines (QPM and non‐QPM)	Amegbor et al. (2022)
PVA (μg g^−1^)	High‐performance liquid chromatography (HPLC)	8.0–17.4	130 inbred lines	Menkir et al. (2015)
	HPLC	3.6–15.3	100 F1 hybrids along parents	Udo et al. (2023)
	Ultra‐Performance Liquid Chromatography (UPLC)	0.7–13.0	161 inbred lines	Garoma et al. (2024)
	HPLC	11.0–28.4	21 maize inbred lines	Menkir et al. (2024)
Fe (mg kg^−1^)	—	9.6–63.2	1814 maize germplasms evaluated	Bänziger and Long (2000)
	Atomic Absorption Spectrophotometer (AAS)	11.3–60.1	30 diverse maize genotypes	Prasanna et al. (2011)
	AAS	12.2–36.7	22 tropical maize inbred lines	Queiroz et al. (2011)
	AAS	16.3–20.4	18 hybrids generated from the line × tester crosses	Akhtar et al. (2023)
	AAS	7.1–58.4	77 single‐cross hybrids	Goredema‐Matongera et al. (2023)
Zn (mg kg^−1^)	—	12.9–57.6	1814 maize germplasms evaluated	Bänziger and Long (2000)
	AAS	15.1–53.0	30 diverse maize genotypes	Prasanna et al. (2011)
	AAS	17.5–42.0	22 tropical maize inbred lines	Queiroz et al. (2011)
	AAS	18.4–23.8	18 hybrids generated from the line × tester crosses	Akhtar et al. (2023)
	AAS	10.7–57.8	77 single‐cross hybrids	Goredema‐Matongera et al. (2023)

The high genetic variability of essential micronutrients such as Zn in QPM genotypes may be attributed to the influence of *o2* and other endosperm modifier genes (Maqbool et al. 2021). The *o2* gene reduces zein synthesis while increasing other protein fractions, such as glutelins, albumins, and globulins, which are known to bind Zn in the maize endosperm (Hindu et al. 2018). Zn plays a significant role in the biosynthesis of tryptophan (Hindu et al. 2018). Chakraborti, Prasanna, Hossain, and Singh (2011) reported that the concentration of Zn in QPM hybrids was greater than that in non‐QPM genotypes. However, not all QPM germplasms exhibit high kernel Zn concentrations; some non‐QPM inbred lines, hybrids, and OPVs also present high Zn contents (Prasanna et al. 2020). Among the 923 lines analyzed for a GWAS regarding Zn content, Hindu et al. (2018) reported that only 31 were QPM or had a QPM background, with 33.3% of those having Zn values exceeding 30 μg g^−1^ dry weight (DW). Conversely, 19.9% of the 892 non‐QPM lines in the panel presented Zn values higher than 30 μg g^−1^ DW, with approximately 6% surpassing the breeding target of 33 μg g^−1^ DW. These findings indicate the significant potential for developing high Zn contents alongside improved‐quality protein through biofortification.

Understanding and exploiting the relationships among grain yield and agronomic and nutritional traits can enhance breeding programs by allowing for more efficient selection processes. This strategy is crucial for improving yield and nutritional quality traits in agricultural production. Many association studies have been conducted to indirectly select yield and quality traits. Ngaboyison et al. (2012) reported that the correlation coefficient between tryptophan content and grain yield was not significant under optimum or random stress but was significant and negative under low nitrogen stress conditions. Bänziger and Long (2000) reported that the grain yield and mineral concentrations of Fe and Zn were negatively correlated. They also noted the difficulty of simultaneously improving both the grain yield and mineral concentration. Although none of the connections between Fe and Zn had significant negative impacts on grain yield, Chakraborti, Prasanna, Hossain, et al. (2011) reported a positive and significant correlation between these two elements. This suggests that it may be possible to enhance both minerals simultaneously without compromising grain yield. Prasanna et al. (2011) reported that the correlations between Zn and Fe did not significantly differ. This study revealed that trait correlations depend on genotype. To improve nutritional effectiveness, it may be preferable to biofortify maize for many nutrients instead of doing each in a separate breeding program because nutrients work together synergistically to improve nutritional effectiveness (Pixley et al. 2011). For example, in comparison with non‐QPM genotypes, QPM has been demonstrated to increase bioavailability and facilitate the absorption of Zn (Prasanna et al. 2020).

### 5.2 Hybrid Breeding and Evaluation

Hybrid breeding is a useful technique for developing new cultivars with desired traits. Although various population improvement procedures exist, developing nutritionally enriched varieties through hybridization is significantly influenced by several factors, including resource availability, genetic variability, breeders' expertise, and high‐throughput phenotyping tools (Maqbool et al. 2018; Goredema‐matongera et al. 2021). Different breeding schemes for nutritionally enriched tropical maize varieties have been conducted at international institutes in collaboration with other stakeholders in the public and private sectors. Notably, the global HarvestPlus Challenge Program has made substantial investments in international institutes such as IITA and CIMMYT to increase PVA and Zn content in maize (Bouis et al. 2024; Gedil et al. 2024).

Recurrent and pedigree selection and the conversion of elite normal maize into provitamin A and QPM are some of the breeding methods employed in maize biofortification. Based on recurrent selection, the breeding population can begin by inter‐mating popular landraces or introducing genotypes with superior nutrient concentrations, followed by selecting the best progenies and repeating the process until stable and optimum or high nutrient concentrations are achieved. This can be achieved by making a backcross with the recurrent parent to restore the desired traits in the original cultivar after crossing an improved donor parent (Goredema‐matongera et al. 2021). Through backcrossing techniques, white normal maize is converted into yellow and/or orange genotypes to improve the PVA concentration (Maqbool et al. 2018), and non‐QPM is converted into QPM (Maqbool et al. 2021). CIMMYT maize research program effectively converted normal populations into QPMs, focusing on yield, kernel modification, kernel appearance, and rapid drying. Once an adequate gene pool of elite QPM lines was generated, QPM breeding at CIMMYT, IITA, and NARS focused primarily on pedigree breeding to develop new inbred lines from QPM × QPM crosses, aiming to establish new segregating families (Krivanek et al. 2007). In addition, new inbred lines are sometimes developed from broad‐based populations via the same process (Raman and Prasanna 2014).

Maize, as a cross‐pollinated crop, requires the development of hybrids via parental lines from diverse heterotic groups. These groups should be resistant or tolerant to biotic and abiotic stresses and should also be nutritionally enriched. In maize biofortification, hybridization involves the development of inbred lines that have good yield and agronomic performance, and an acceptable concentration of essential nutrients. Once these inbred lines are established, they are crossed to create improved hybrids, including single, three‐way, and double‐cross hybrids. The value of an inbred line in a hybrid combination depends on its effective combining ability with other lines to produce high‐performing hybrids. Therefore, the selected inbred lines must undergo thorough screening for general combining ability (GCA) and specific combining ability (SCA), as well as analysis of gene action related to target nutrients and other important agronomic traits (Menkir et al. 2015). Various studies have assessed the potential of Zn, PVA, and QPM inbred lines for hybrid breeding by evaluating their combined ability estimates and genetic control of various traits. Amegbor et al. (2023) reported a predominance of additive genes influencing tryptophan content when 10 non‐QPM and 23 QPM lines were used in combination with four elite testers (two QPM lines and two non‐QPM lines). Azmach et al. (2021) experimented with 24 genetically diverse yellow and orange endosperm maize inbred lines to analyze their ability to combine carotenoids and found significant GCA and SCA effects for most carotenoids and provitamin A contents; however, the GCA effects were greater than the SCA effects, reflecting the main contribution of additive gene action. Both additive and nonadditive gene effects are important in controlling Zn and Fe; however, GCA effects outweigh SCA effects (Mageto et al. 2020; Akhtar et al. 2023). Moreover, Udo et al. (2023) reported that additive gene effects controlled the accumulation of PVA and Fe, whereas both additive and nonadditive gene effects controlled the inheritance of Zn and grain yield, identifying inbred lines that could be used to develop hybrids and synthetics that combine high grain yield with high single or multiple micronutrients. They also indicated the feasibility of enriching maize with multiple micronutrients without compromising grain yield. The prevalence of GCA effects in nutritional trials indicates that additive gene effects are more important than nonadditive or epistatic gene effects for these quality traits. After promising genotypes are identified, they have to be tested in various target environments to assess genotype × environment interaction (G × E) and their impact on micronutrient expression (Bouis and Saltzman 2017). Significant effect of G × E interaction for PVA (Mengesha et al. 2019) for Fe and Zn concentrations (Prasanna et al. 2011) has been reported in diverse maize populations.

### 5.3 Agronomic Practices

Agronomic practices for biofortification are straightforward and economical, but environmental factors, nutrient types, and application methods require careful consideration (Shahzad et al. 2021). Zn is one of the most important micronutrients for increasing maize yields and creating nutrient‐dense grains (Garg et al. 2018). Soil fertilization, foliar spraying, and seed dressing are all successful agronomic techniques for maize Zn biofortification (Figure 3). Mutambu et al. (2023) reported that applying Zn fertilizer significantly increased Zn levels in maize tissue and grain, resulting in increases of 17% in maize grain yield and 25% in grain Zn concentration. Consequently, yields of up to 1 t ha^−1^ and grain Zn concentrations of 7.19 mg kg^−1^ were noted compared with those of controls without Zn (Mutambu et al. 2023). Plants can absorb more zinc when soil Zn fertility is increased, and this zinc is subsequently transferred to grains and other areas (Qurban et al. 2022). There are several different types of Zn fertilizers, but the most common are zinc sulfate (ZnSO4) and zinc oxide (ZnO). The optimal application rate of Zn fertilizer for maize to increase grain yield and Zn levels depends on the soil conditions and target Zn concentrations in the grains. Soil organic matter, pH, available soil zinc, organic input applications, and the rates and techniques of N, Zn, and Fe applications are important determinants of grain Zn. Compared with no Zn application, the annual application of 10.0 kg Zn ha^−1^ led to improved grain size and total Zn concentrations in maize and wheat (Bhardwaj et al. 2022). In general, combining soil and foliar applications of Zn significantly increases grain yield and grain Zn concentration (Kihara et al. 2024).

### 5.4 Mutation Breeding

In plant breeding, mutation is a useful technique for generating genetic diversity that can increase grain yield, nutritional value, disease resistance, and other beneficial traits. Mutagenesis has a long history in the genetic enhancement of crop plants, targeting both quantitative and qualitative traits. Genetic change is ultimately derived from the process of altering the nucleotide sequence of an organism's DNA. Mutagens such as X‐rays and gamma rays are used to treat seeds, and chemical agents such as ethyl methane sulfonate (EMS) are important for causing random DNA changes in various crops (Goredema‐matongera et al. 2021). The use of strategies that involve both spontaneous and induced mutants allows the characterization of complex gene expression systems that integrate carbohydrate, amino acid, and storage protein metabolism during endosperm growth and development (Hartings et al. 2012). Mutants associated with the biosynthesis of carotenoids, including *y1*, *vp2*, *vp5*, *vp7*, *vp9*, *w3*, and *y9* (Maqbool et al. 2018), as well as a number of endosperm gene mutations associated with reducing zein synthesis, have different biochemical effects on tryptophan and lysine contents, such as *o2*, *floury2 (fl2)*, *opaque7 (o7)*, *opaque6 (o6), floury3 (fl3)*, *mucronate (Mc)*, *defective endosperm (De‐B30)*, *opaque7749*, *opaque7455 (o11)*, and *opaque16* (Hartings et al. 2012).

### 5.5 Molecular Marker Applications for Maize Biofortification

The development of a biofortified variety involves identifying and transferring desirable genes from a donor to a superior agronomic recipient parental line via the use of molecular breeding tools (Sheoran et al. 2022). Among the various molecular breeding methods, MAS plays a crucial role in enhancing the efficiency of selection. This approach has accelerated the development of new biofortified varieties that not only have high yield potential but also feature superior nutritional quality traits (Prasanna et al. 2020). MAS is an indirect selection process in which the desired trait is selected through specific markers, which can be morphological, biochemical, or DNA/RNA markers (Goredema‐matongera et al. 2021). Marker‐assisted foreground selection (MAFS) uses DNA markers to identify individuals who carry the gene(s) of interest from a donor parent, whereas marker‐assisted background selection helps speed up the recovery of the recurrent parent's genome in backcross lines. For example, in QPM breeding, both foreground and background MAS can be effectively utilized to select the *o2* phenotype while ensuring maximum recovery of the recurrent parent (Shankar 2016). Babu et al. (2005) reported that a two‐generation marker‐based backcross breeding approach could be sufficient for incorporating the *o2* gene, along with phenotypic selection for kernel modification in maize inbred lines. The use of marker‐assisted backcrossing (MAB) greatly enhances selection efficiency, particularly for traits controlled by recessive alleles such as QPM, which are challenging to manage with conventional methods (Collard and Mackill 2008). Molecular markers based on functional polymorphisms within *PSY1*, *LcyE*, and *CRTRB1* provide a quick means of increasing PVA carotenoid concentrations in maize (Babu et al. 2013). MAB can expedite the introgression of favorable alleles of *LCYE* and *CRTRB1* into tropical materials from temperate donors, optimizing the breeding process for PVA content (Babu et al. 2013; Yang et al. 2018). The other important MAS breeding approach to improve a population is marker‐assisted recurrent selection (MARS) (Gedil et al. 2024). In two synthetic maize populations (heterotic group A (HGA) and heterotic group B (HGB)) in two cycles of MARS, HGA increased β‐carotene, PVA, and total carotenoids content by 40%, 30%, and 36%, respectively, while HGB population improved α‐carotene and β‐cryptoxanthin content 20% and 5%, respectively (Gedil et al. 2024). In general, CIMMYT and IITA breeders have developed several tropical maize lines and populations that have improved PVA content through MAS (Menkir et al. 2017).

Numerous molecular markers associated with the *o2* gene have been identified and shown to be beneficial for MAS (Gupta and Saha 2009). Several genes regulating amino acid content have also been identified (Tandzi et al. 2017). CIMMYT developed simple sequence repeat (SSR) markers such as *phi057*, *phi112*, and *umc1066*, which are located within the *o2* gene on the short arm of chromosome 7 and effectively distinguish between the *O2* and *o2* alleles (Bantte and Prasanna 2003). Among these genes, *phi057* and *Umc1066* are codominant markers, whereas *phi112* functions as a dominant marker. Bantte and Prasanna (2003) distinguished 30 unique SSR alleles to differentiate QPM inbred lines and recognized those carrying the *o2* mutation via the *phi057* SSR primer. Molecular markers have also been used to examine genetic diversity and uncover heterotic patterns within QPM inbred lines (Wegary et al. 2019). This approach could augment the diversity of QPM germplasms by integrating maize germplasms from various tropical maize breeding programs.

Through molecular dissection, genes or QTLs correlated with micronutrients such as β‐carotenoids, Fe, Zn, and essential amino acids in maize have been identified. Identifying QTLs for nutritional content in maize represents a strategic focus for MAS aimed at creating mineral‐enriched maize genotypes (Qin et al. 2012; Maqbool and Beshir 2019). In QTL mapping studies for Fe and Zn contents, several genomic regions harboring multiple QTLs have been identified on various chromosomes. Qin et al. (2012) pinpointed three stable QTLs that can be used for improving Zn and Fe concentrations. Jin et al. (2013) identified five significant QTLs enhancing grain Zn and Fe contents in an F2:3 mapping population through QTL mapping and meta‐analysis studies. Zhang et al. (2017) examined QTLs for Zn, Fe, copper (Cu), and manganese (Mn) concentrations in maize kernels, both in a single environment and across several environments, and discovered 64 and 67 QTLs for single and multiple environment evaluations, respectively. A GWAS involving 923 CIMMYT maize inbred lines found 20 SNPs associated with kernel Zn and 26 with Fe content (Hindu et al. 2018). Another study identified 11 candidate genes linked to Fe concentrations. Among these genes, ZmNAC78 was the only gene that consistently showed higher expression in lines with higher Fe concentration in their kernels (Ashraf and Bakirbas 2024). To explore the application of ZmNAC78 in breeding, genotypes were grouped into haplotype 1 (Hap1) and haplotype 2 (Hap2). The abundance of ZmNAC78 and Fe concentrations was higher in Hap1 than in Hap2 (Du and Li 2024). Hap1 varieties also had greater grain yield and Fe concentration, suggesting ZmNAC78 gene potential in maize Fe biofortification (Ashraf and Bakirbas 2024; Du and Li 2024).

### 5.6 Genetic Engineering

When the gene pool and its wild relatives have little genetic variation, transgenic technology can be useful for improving the trait of interest (Shahzad et al. 2021). Genetic engineering makes it possible to improve the nutritional quality of maize through the introduction of particular genes that increase the levels of vital nutrients such as proteins, vitamins, and minerals (Maqbool et al. 2018; Goredema‐matongera et al. 2021). Transgenes have been incorporated into the maize genome via various techniques, including Agrobacterium‐mediated transformation and biolistic transformation (particle bombardment), aimed at developing “biofortified” maize varieties with improved nutritional value to address malnutrition issues, especially in areas where maize is a staple food (Yadava et al. 2017). The bacterium *Agrobacterium tumefaciens* , whose DNA (T‐DNA) is naturally introduced into plant cells, was used in this method. When Agrobacterium infects plant tissue, researchers alter the T‐DNA to incorporate the desired transgene, which makes it easier for the transgene to integrate into the maize genome (Que et al. 2014).

Genetically modified (GM) maize varieties with significantly enhanced commercial traits have been developed recently due to major advances in plant biotechnology. Biotech maize, also known as GM maize cultivars with resistance to both biotic and abiotic stresses, including pests, herbicides, and drought, is widely grown around the world, with significant production in many countries. Numerous studies have documented the successful creation of biofortified transgenic maize varieties. For example, genetic engineering aimed at altering Zn uptake, root remobilization, xylem loading and transport, grain remobilization, and kernel sequestration offers the possibility of increasing the kernel Zn concentration (Maqbool and Beshir 2019). The lysine content of the maize endosperm protein has increased by 15%–20% as a result of researchers using RNA interference (RNAi) technology to reduce the levels of 22‐kDa and 19‐kDa α‐zein proteins (Maqbool et al. 2021). By effectively changing their metabolic pathways, transgenic techniques offer a reliable way to increase the levels of different vitamins in crop plants (Maqbool et al. 2018). This creative approach has the potential to greatly improve the nutritional value of food, which would benefit consumers. Although many studies have confirmed the safety of GM crops for human and animal consumption as well as their effects on the environment, debates about the biosafety and public acceptance of GM food crops are still common in the scientific and political communities.

### 5.7 Genome Editing

Genome editing (GE) represents a groundbreaking biotechnological advancement that allows significant enhancement of the nutritional content of maize and other crops, thereby improving food and nutrient security for future generations. This molecular biology method modifies plant DNA to introduce or enhance desirable traits. To change particular DNA sequences, molecular tools such as CRISPR–Cas, TALENs, and ZFNs act as molecular scissors (Shahzad et al. 2021). These tools show great promise for enhancing nutritional quality traits in various crop species. For example, “golden rice,” enriched with beta‐carotene, exemplifies the effectiveness of genetic modification in enhancing provitamin A biosynthesis (Chen et al. 2024). GE technology has successfully increased the mineral content in crops, such as by editing OsNAS genes in rice and wheat to increase iron and zinc levels and address micronutrient deficiencies (Chen et al. 2024). The modification of the *PSY1, Crtl*, and *LCYB* genes in maize has resulted in “golden maize” increasing provitamin A production (Sobrino‐Mengual et al. 2024). GE improves essential amino acid levels in cassava and lysine levels in maize (Hasan 2024). Targeted mutagenesis of the *OsAAP6* and *OsAAP10* genes in rice enhances grain quality while increasing protein content (Wang et al. 2020). Moreover, this technology effectively reduces antinutritional components such as phytic acid in soybeans, improving the bioavailability of Fe and Zn (Song et al. 2022).

## 6 Phytic Acid Content and Bioavailability of Micronutrients in Maize

Phytate, known as myo‐inositol‐1,2,3,4,5,6‐hexakisphosphate (PA), is a common compound found in plant seeds that serves as a storage house of phosphorus (P) (Korge et al. 2025). It represents about 75% of the total phosphorus in mature seeds (Raboy 2009; Wu et al. 2009). Following synthesis during seed development, it accumulates and deposits as mixed “phytate” or “phytin” salts primarily of potassium (K) and magnesium (Mg) (Raboy 2020). These salts may also contain Fe, Zn, and other mineral cations (Raboy 2020). Phytate is often known as an antinutrient because it can form complexes with proteins and certain essential micronutrients, such as Fe and Zn, significantly reducing their bioavailability (Kumar et al. 2021). In addition to its negative effects, it also plays a beneficial role for humans as an antioxidant and has antidiabetic and antibacterial effects (Bloot et al. 2023; Korge et al. 2025). Bioavailability refers to the portion of essential nutrients such as minerals (Fe and Zn) digested, absorbed, and used in metabolism (Kumar et al. 2015; Akhtar et al. 2018). However, the delivery of these elements from maize‐based food products is compromised due to high levels of phytates (Nsabimana et al. 2024).

Based on the cultivars and management conditions, the concentration of phytates in maize ranges from 2.77 to 16.70 mg·g^−1^ of seeds (Grases et al. 2017; Nsabimana et al. 2024). The estimated impact of phytates on mineral bioavailability is measured by calculating phytate‐to‐mineral ratios. Phytates/Zinc (PA/Zn) molar ratio < 18 and Phytates/Iron (PA/Fe) molar ratio < 1 are considered as adequate for optimal absorption of these minerals (Gibson et al. 2010). However, maize exceeds these molar ratio thresholds, contributing to higher risks of Fe and Zn deficiency among regular consumers of maize and maize‐based products (Gallego‐Castillo et al. 2021). To reduce phytate levels in cereals, several strategies have been implemented. These strategies include genetic enhancements through different breeding and genetic engineering approaches. These methods are being explored as effective alternatives to increase mineral content and bioavailability in cereals (Shahzad et al. 2014). Several research results revealed significant genetic variability in PA level and PA/Fe and PA/Zn molar ratios within maize germplasms. For example, Šimić et al. (2009) reported notable genetic variations in the PA/Fe and PA/Zn molar ratios in 294 F4 inbred lines of a maize population. Queiroz et al. (2011) found considerable genetic variability in the PA/Zn molar ratio (18.0–43.5) and the PA/Fe molar ratio (16.3–45.5) among 22 tropical maize inbred lines. These studies indicate the potential for breeding strategies to improve Fe and Zn bioavailability in maize germplasm.

Food processing methods, such as enzymatic and heat treatments like cooking and baking, are used to reduce phytates. However, each method has its limitations. For example, enzymatic methods can be costly (Nsabimana et al. 2024). In addition, phytates are heat‐stable, which means that heat treatments like cooking and baking have limited effectiveness in reducing their levels (Nsabimana et al. 2024). Traditional processes such as fermentation and malting under controlled conditions have been other effective methods to degrade phytates and improve mineral bioavailability in various crops (Atuna et al. 2022). During fermentation, either microbial phytase production or the activation of endogenous phytase can effectively hydrolyse phytates, thereby enhancing the bioavailability of minerals (Castro‐Alba et al. 2019). Phytate content reduction through the soaking, germination, and fermentation processes in maize reached up to 85% (Nsabimana et al. 2024).

## 7 Challenges of Biofortified Maize Development and Adoption

In developing nations where maize is a staple crop, the development and adoption of biofortified maize enhanced with lysine, tryptophan, PVA, and minerals holds great promise for lowering protein‐energy malnutrition and diseases related to micronutrient deficiencies. Despite the numerous biofortified maize varieties that have been developed and released in various developing countries, there are many challenges in the wider adoption of biofortified varieties (Tandzi et al. 2017; Maqbool et al. 2021). Limited research on biofortification, along with weak research infrastructures and inadequate laboratory facilities for molecular breeding, as well as poor extension mechanisms, are major challenges in the research and dissemination of biofortified maize in many developing countries.

### 7.1 Challenges Related to QPM Development and Adoption

The QPM has recently encountered significant challenges in international and regional research institutes. First, there is a lack of funding for QPM research in developing countries, where the nutritional benefits of QPM could have the most substantial impact. This financial constraint hampers the development and dissemination of QPM varieties suited to local conditions. Since the nutritional quality of QPM varieties is controlled by a recessive mutant allele of the *o2* gene, deterioration or dilution due to foreign pollen of non‐QPM varieties planted adjacent to QPM is also a serious concern for producing quality seeds (Maqbool et al. 2021).

There are considerable challenges in effectively communicating the advantages of QPM to farmers, who may be unaware of its nutritional benefits compared with normal/non‐QPM maize varieties. This lack of awareness can result in hesitance to adopt QPM varieties (Jambo et al. 2024). Misconceptions about the low yield of QPM, alongside uncertainties regarding how the market will respond to these varieties, demotivate growers from adopting QPM varieties. Such apprehensions can further prevent farmers from transitioning to QPM.

The lack of cooperation among researchers, policymakers, and the farming community is a significant obstacle to the dissemination of QPM varieties (Jambo et al. 2024). A coordinated effort is necessary to create policies that support the research and dissemination of QPM and address both the educational needs of farmers and the logistical challenges of integrating QPM into existing agricultural systems. Without enhanced partnerships and strategic investments, the full potential of QPM in improving food security and nutrition in developing regions remains unrealized.

### 7.2 Challenges Related to Provitamin A Development and Adoption

The development of provitamin A maize in Africa faces several challenges. The provitamin A carotenoids are sensitive to light and air. Carotenoids are reduced when maize grains are sun‐dried or heated to high temperatures (Shrestha and Karki 2015). Certain cooking techniques can cause the loss of provitamin A carotenoids in food. The best methods for preserving provitamin A are boiling and steaming; roasting, frying, and drying can result in considerable losses. Therefore, breeders should target not only the concentration of nutrients in preprocessed kernels but also the genetic variation of compounds in post‐processed kernels (Gedil et al. 2024).

A negative perception of orange and yellow maize is common in African countries. Many people associate yellow and orange maize‐based foods with poverty, viewing them as suitable only for food aid during emergencies (Groote et al. 2011). Despite recent years, the acceptance of orange and yellow maize varieties has improved in a few countries; still, it needs some efforts to adopt the varieties in all SSA countries. Moreover, there is a general lack of awareness about the nutritional benefits of PVA maize among both farmers and consumers (Kondwakwenda et al. 2018). Educational initiatives are needed to inform stakeholders about how PVA biofortified maize can help alleviate vitamin A deficiency, especially in children and pregnant women.

### 7.3 Challenges Related to the Development of Zn‐Enriched Maize Genotypes

The development of Zn‐enriched maize varieties is a multifaceted endeavor that faces complicated challenges. The availability of Zn in the soil is influenced by the soil composition, pH value, and organic matter content (Saleem et al. 2022). Low soil Zn levels can severely impede the uptake of this essential micronutrient by maize plants, highlighting the need for soil management practices that optimize nutrient availability. Regular soil testing and amendments may be needed to ensure sufficient Zn levels for optimal plant growth. Phytic acid, a common component found in maize grains, can bind to Zn and inhibit its absorption in the human digestive system. This complex interaction complicates efforts to not only increase the Zn concentration in the grains but also ensure that it is bioavailable to consumers (Akhtar et al. 2018). Understanding these biochemical interactions is crucial for developing maize varieties that are both rich in Zn and easily utilized by the body. Successfully addressing these interrelated challenges requires a holistic approach that maintains high yields while increasing the Zn concentration in maize grains. Through collaborative efforts among breeders, soil scientists, nutritionists, and the agricultural community, it is possible to create maize varieties that not only meet dietary needs but also contribute to sustainable agricultural practices.

## 8 Conclusions and Recommendations

Biofortification is still an emerging technology used to enhance the nutritional quality of maize for addressing malnutrition problems in SSA. Biofortification approaches such as soil fertilization, foliar spraying, seed dressing, hybridization, mutation, molecular breeding methods, genome editing, and genetic engineering are crucial for developing nutritionally enriched maize varieties. Screening of inbred lines via phenotypic and existing molecular markers while conducting chemical analyses of carotenoid, Zn, and tryptophan contents is important to achieve our targets. This step is crucial for verifying nutritional levels in promising genotypes. Such efforts should be combined with the continual refinement of heterotic pools, which is essential for the sustained development and adaptation of biofortified maize varieties. Evaluating newly developed biofortified varieties across locations can help to assess genotype × environment interaction (G × E) and their impact on micronutrient expression. Creating a strong monitoring and evaluation system to track the adoption rates and impacts of biofortified maize will be invaluable for fine‐tuning strategies and showing success for donors, policymakers, and stakeholders.

## Funding

The authors have nothing to report.

## Conflicts of Interest

The authors declare no conflicts of interest.

## Data Availability

Data sharing not applicable to this article as no datasets were generated or analysed during the current study.
